# Barriers to Buprenorphine Treatment Among People Experiencing Homelessness: A Qualitative Study from the Provider Perspective

**DOI:** 10.1007/s11524-025-00967-y

**Published:** 2025-03-27

**Authors:** Carmen L. Masson, Kelly R. Knight, Emily A. Levine, Joseph A. Spillane, Ya Chi Angelina Liang, Leslie W. Suen, Maggie M. Chen, Barry Zevin, Robert P. Schwartz, Phillip O. Coffin, James L. Sorensen

**Affiliations:** 1 Department of Psychiatry and Behavioral Sciences, University of California, San Francisco, San Francisco, CA USA; 2 Department of Humanities and Social Sciences and Center for Vulnerable Populations, University of California, San Francisco, San Francisco, CA USA; 3 Department of Medicine, University of California, San Francisco, San Francisco, CA USA; 4 Friends Research Institute, Los Angeles, CA USA; 5 San Francisco Department of Public Health, San Francisco, CA USA

**Keywords:** Buprenorphine, Barriers, Homelessness, Opioid use disorder, Clinicians, Community-based providers

## Abstract

People experiencing homelessness (PEH) face a high risk of opioid-related deaths, yet there is limited qualitative data on the barriers encountered when accessing buprenorphine treatment for opioid use disorder (OUD). To address this gap, we interviewed 28 clinicians, outreach workers, and administrators from organizations serving PEH with OUD. Our goal was to understand the barriers and facilitators at the patient, clinic, and institutional levels and gather recommendations for improvement. Interviews, conducted via Zoom and analyzed through thematic analysis, revealed several barriers. At the patient level, themes related to barriers included knowledge and experience (e.g., limited knowledge about buprenorphine options; rejection of buprenorphine due to prior experience with precipitated withdrawal); concerns about the medication and its administration (e.g., distrust of injectable medications; concerns about treatment control, and a prolonged informed consent process for extended-release injectable buprenorphine); and challenges due to homelessness (e.g., identification requirement to access medication at pharmacies, difficulties managing buprenorphine while unsheltered). At the clinic level, themes centered around staffing (e.g., lack of training and experience in treating PEH and staffing shortages) and health care-related stigma (e.g., discriminatory attitudes toward PEH with OUD). Institutional-level themes included state-regulatory factors (e.g., practice regulations limiting clinical pharmacists’ ability to prescribe buprenorphine) and access factors (e.g., stigmatization of buprenorphine prescribing, limited low-barrier buprenorphine access, and care system complexity). Recommendations included educational programs for patients and clinicians to increase understanding and reduce stigma, integrating buprenorphine treatment into non-traditional settings, and providing housing with treatment.

## Introduction

Opioid overdose is a major cause of death among people experiencing homelessness (PEH) [1]. Nationally, overdose deaths from synthetic opioids, primarily fentanyl, continued to increase in 2023 [2], with fentanyl often combined with other illicit substances in non-fatal overdoses [3]. Homelessness and unstable housing create barriers to entering and adhering to substance use disorder treatment, as daily survival needs, like food and shelter, competed with medication access and adherence while homeless [4, 5]. PEH also face challenges like loss, seizure, or theft of belongings, including medications, and may not be able to leave belongings to travel to clinics or pharmacies. Injectable extended-release buprenorphine (XR-buprenorphine) may benefit PEH who have difficulty storing medications, following daily medication regimens, or experiencing frequent treatment disruptions [6–8].

Despite the availability of buprenorphine in various forms, such as buccal films and sublingual tablets, PEH often struggle with adherence to sublingual formulations, resulting in low retention rates [8]. XR-buprenorphine, with its fixed dosing schedule (1 or 6 months), may offer a more feasible alternative for those facing adherence challenges, including PEH [9]. Recent studies suggest that XR-buprenorphine may reduce non-fatal overdose rates compared to daily sublingual buprenorphine or methadone, making it a promising option for PEH [10]. However, since XR-buprenorphine is a new formulation with limited data on its use among PEH, it is essential to understand the perspectives of providers who manage this population. Providers serve as the gatekeepers to buprenorphine treatment, and exploring the barriers they encounter in delivering care to PEH with OUD can offer valuable insights. By identifying these barriers, we can better inform the design of targeted interventions to improve both treatment access and retention among this vulnerable group.

Few studies have explored the perspectives of providers on the barriers that PEH face in accessing, initiating, and continuing buprenorphine treatment. To fill this gap, we studied clinicians’ and community-based service providers’ perspectives on patient, clinic, and institutional-level barriers and facilitators that influence buprenorphine treatment uptake among PEH with OUD.

## Methods

### Participants

Similar to the national trend, fentanyl-related overdose deaths among PEH in San Francisco rose during the first year of the COVID pandemic. With its unique demographic and public health challenges, San Francisco was an ideal setting for this study. Participants were recruited from three organizations in San Francisco, California, that provide healthcare and support services to PEH with OUD, including primary care, pharmacy, street-based, low-threshold buprenorphine access programs, syringe access, and overdose prevention. Using purposive sampling, program directors and medical directors identified eligible staff, and invitations were sent via email. Eligibility criteria included being at least 18 years old, English-speaking, and having at least 1 year of experience as a clinician (e.g., primary care clinician, clinical pharmacist, nurse practitioner, registered nurse) or community support service staff or administrator (e.g., outreach worker, program director/administrator, training coordinator) serving PEH with OUD. Thirty-four potential participants were successfully contacted; of these, 3 declined to participate, 1 did not meet eligibility criteria, and 2 were used to pilot test the interview guide, which resulted in a study sample of *N* = 28. Half of the sample (*n* = 14) consisted of clinicians working in government-funded health or social programs under the City and County of San Francisco, while the other half were community-based support staff or administrators from two large non-profit harm reduction programs. We ceased interviewing when we reached thematic saturation [11].

### Data Collection

The Consolidated Framework for Implementation Research (CFIR) guided the interview guide design, data analysis, and interpretation [12]. The semi-structured interview guide covered CFIR’s five domains, intervention characteristics, outer setting, inner setting, characteristics of individuals, and process focusing on the uptake and delivery of buprenorphine treatment for PEH. Outer setting questions focused on the broader environment of homelessness and other external factors. Inner setting questions addressed buprenorphine use in clinical and street medicine settings. Individual characteristics questions explored PEH motivations and experiences, as well as providers’ experiences offering and managing buprenorphine treatment for PEH. Process-related questions examined implementation factors influencing buprenorphine initiation and use among PEH. The interview guide focused on patient-, clinic-, and institutional-level barriers and facilitators to buprenorphine treatment for PEH, emphasizing both sublingual and XR-buprenorphine formulations. Interview guides included open-ended questions such as “What do you see as the goals of buprenorphine treatment for people experiencing homelessness?”. Interviews lasting approximately 90 min were conducted between March 2021 and July 2021 over Zoom by two women research team members (C.L.M. or J.S.).

Before the interview, the interviewer obtained informed consent and collected demographic information. Probes were used to encourage further responses or expand on participant ideas. Key statements were rephrased for verification of accurate interpretation of participant responses. Interviews were audio recorded and professionally transcribed. Participants received a $50 gift card as compensation. The Institutional Review Board of the University of California, San Francisco, approved research procedures (IRB# 19–28,720).

### Data Analysis and Framework

The analysis used a thematic approach based on modified grounded theory methodologies [13]. Our analysis was also guided by the constructs of the CFIR [12], which allowed for the emergence of new themes/codes from the data. We developed a codebook that was applied to each transcribed interview. The codebook was developed through an iterative, team-based process in which transcription was read by multiple team members trained in qualitative research methods (Y.C.A.L; J.A.S.; C.L.M.; L.W.S.; K.R.K.) and discussed in a series of lengthy Zoom-based meetings over several months. The team identified broad content topics in these meetings. Once all transcripts were read and discussed, we held an additional series of team-based meetings to reduce the broad content areas into codes, which formed the preliminary codebook. Twelve interviews were double coded independently by two team members (Y.C.A.L and J.A.S.), and discrepancies between uses of codes were discussed between independent coders to ensure inter-rater agreement. After double-coded transcripts were finalized, each remaining transcript was assigned to one primary coder and one secondary coder who reviewed the primary coder’s work and discussed any discrepancies. After all interviews were coded, team members transferred all coded content into Dedoose (Version 9.0.17), a web application for managing, analyzing, and presenting qualitative and mixed-method research data [14]. This analysis focused on the “Barriers” code and other relevant codes, in line with the study’s goal of identifying obstacles to buprenorphine access, initiation, and use among PEH, along with recommendations for addressing them. During team-based analysis meetings discussing data relevant to these codes, the topology of patient, clinic, and institutional levels emerged as salient categories for discussing the locus of barriers and their remediation. Within each domain, specific themes emerged, evidenced by quotes, to describe the study findings.

## Results

### Participant Characteristics

Participants (*N* = 28) reported a mean age of 39.9 years (SD = 8.8); 19 were female, 6 male, and 3 transgender or non-binary. Almost half identified as White (13); 2 participants identified as Asian, 2 as Black/African American, 10 as other or multiple races, and 1 declined to answer. Fourteen were clinicians (7 registered nurses, 3 nurse practitioners, 2 pharmacists, and 2 physicians), 7 were outreach workers, and 7 were program directors/administrators.

Participants were prompted by the interview guide to report on perceived barriers and facilitators to buprenorphine treatment experienced by PEH at the patient, clinic, and health institutional levels. At the patient level, themes related to barriers included *knowledge and experience* (e.g., limited knowledge about buprenorphine options; rejection of buprenorphine due to prior experience with precipitated withdrawal); *concerns about the medication and its administration* (e.g., distrust of injectable medications; concerns about treatment control, and a prolonged informed consent process for XR-buprenorphine); and *challenges due to homelessness* (e.g., identification requirement to access medication at pharmacies, difficulties managing buprenorphine while unsheltered). At the clinic level, themes centered around *staffing* (e.g., lack of training and experience in treating PEH and staffing shortages) and *health care-related stigma* (e.g., discriminatory attitudes toward PEH with OUD). Institutional-level themes included *state-regulatory factors* (e.g., practice regulations limiting clinical pharmacists’ ability to prescribe buprenorphine) and *access factors* (e.g., stigmatization of buprenorphine prescribing, limited low-barrier buprenorphine access, and care system complexity). Quotes are displayed in the body of the text, and additional exemplifying themes for patient-level, clinic-level, and institutional-level barriers are displayed in Table 1.

**Table 1:** Provider quotes supporting patient, clinic, and institutional-level barriers to buprenorphine treatment

Level	Quotes	Themes
Patient-level	(Q1) [S]ince it’s the pandemic and you have all these homeless [people] in these hotels and they’re safe, and they eat and they’re warm, they’re not cold and they’re on a mattress and they get to watch TV, they get to leave when they want to, they get to come back when they want to. It’s like housing. It’s probably a good time to talk to the medical team or the homeless outreach team to get buprenorphine and to introduce it to the clients” (Support Service Administrator)	Knowledge and information Challenges related to homelessness
Clinic-level	(Q2) “I struggle with the tension between Sublocade as [sufficient] enough and prescribing Suboxone on top of that for people. And I have prescribed Suboxone on top of Sublocade, just to help with that feeling of waxing and waning cravings, particularly, at the end of Sublocade. So I think that’s one thing I struggle with, [a fixed dosage] of Sublocade. (Clinician)	Staffing
Institutional-level	(Q3) “…An existing client who either had a gap in care or had fallen out of care… what we’ll do in those situations is look at the last person who prescribed and try to get a hold of that person…but many times that process can take a really long time. Oftentimes we don’t get a hold of the provider right away and then the client ends up sitting in the pharmacy for hours just waiting to see what the plan is going to be” (Clinician)	Access

### Patient-Level Barriers

Participants reported that a lack of proper information about buprenorphine formulations, and prior experiences with buprenorphine treatment, posed significant barriers at the patient level. Some participants mentioned that misinformation, including unfounded fears about injectable buprenorphine, contributed to reluctance toward XR-buprenorphine. For example, one participant reported: “*It was more of a conspiracy theory type of thing– they’re probably tagging me, I don’t want an injectable*.” (Clinician) They also described that patients are fearful of, or distrust, injectable medications. Related to the theme of *concerns about the medication*, one clinician reported: “*They [PEH] don’t trust prescribed, injectable meds, like vaccines and things like that, same thing for Sublocade*.” (Clinician) Additionally, participants expressed that their patients had heard from friends or other social network members that the buprenorphine injection was painful or that the medication was ineffective.

Related to the theme of *knowledge and information* informing patient decision-making, one clinician reported: “*…there’s a lot of misinformation about buprenorphine…people are like, ‘Oh no, I’m allergic to that, like I got sick when I took that once,’ so [they] are just completely unwilling to consider it based on either their bad experience with it or a friend’s bad experience with it.*” (Clinician).

In contrast, participants also reported that some PEH reject buprenorphine based on their own previous negative experiences, particularly precipitated withdrawal, with either prescribed or non-prescribed buprenorphine, often in the context of fentanyl use. One clinician noted, “…*there are some people who have had those bad experiences with buprenorphine that precipitated withdrawal– they’ve made up their minds, the door is shut*.” (Clinician).

Some participants noted that patients may fear losing control over XR-buprenorphine administration or feel uncomfortable with the inability to stop treatment. Consistent with the theme of *concerns about the medication’s administration*, they reported that patients often avoid medications that reduce their autonomy: “….*Like anything injectable, especially if someone has psych issues, [it] raises a whole bunch of red flags for people because they don’t have control over administering it and there can be some paranoia around that*.” (Clinician).

Another finding that centered around the theme of *concerns about the medication and its administration* was related to the prolonged consent process, especially for XR-buprenorphine. Due to the treatment’s complexity, inflexibility once started, and potential for patient misunderstandings, adequate time is needed for informed consent, often requiring multiple sessions. Additionally, participants highlighted that PEH often experience co-occurring psychiatric disorders, polysubstance use, and severe addictions, which can impair cognitive function and further complicate the consent process. Some participants emphasized that XR-buprenorphine may not be a suitable first-line medication for OUD, particularly for patients with co-occurring psychiatric disorders who may require immediate treatment for those conditions. However, some participants acknowledged that XR-buprenorphine could be beneficial for patients who struggle with appointments or frequently lose their medications.

“…*within our homeless population, we also have a lot of mental health and psych issues, too and most people are dual diagnosis and then just trying to make sure that people are truly informed on what it is… they’re taking. And that means treating their psych condition first so that they’re fully aware of what it entails. That it’s a four-week thing, …and they’re fully informed of what the drug is*.” (Clinician).

Related to the theme of *challenges due to homelessness*, participants emphasized that experiencing withdrawal while homeless without easy access to privacy, a place to safely rest, or a bathroom presents significant challenges. Traditional buprenorphine initiation requires patients to achieve a state of opioid withdrawal, common symptoms of which are chills, stomach pain, aches, diarrhea, and vomiting, often producing extreme discomfort: “*Trying to go through the withdrawal process on the street is particularly hard. You don’t have access to a bathroom, and you’re already uncomfortable being on the street and then having to feel uncomfortable going through withdrawal*.” (Clinician).

Again, related to the theme of *challenges due to homelessness*, participants noted that patients’ living environments, particularly for those who are unsheltered, made it difficult to stay engaged in treatment. They also reported that refraining from substance use or seeking treatment is challenging in environments with a high concentration of others using substances: “*Environment is a big problem…it kind of triggers them back into wanting to use again when they’re in that environment or with those people*.” (Clinician).

Counteracting numerous barriers that we identified related to the theme of *challenges due to homelessness*, efforts to house PEH in non-congregate, “shelter-in-place” hotel rooms early in the COVID-19 pandemic helped address homelessness-related barriers. Participants reported that these housing solutions allowed PEH to stabilize and initiate buprenorphine, overcoming challenges like withdrawal management, storage, and consistent access to prescriptions (Q1). However, many of these housing placements were unfortunately temporary.

“*I’ve had a few clients tell me since COVID, and since they got housed, that it saved their life because they now had a place they could go to and they no longer went to the areas on the street they used to go that would cause them to relapse. So, I don’t have all the triggers anymore and I’ve been able to stay on the buprenorphine longer than I ever have before*.” (Clinician).

### Clinic-Level Barriers

We identified two phenomena that informed buprenorphine initiation and utilization that related to the theme of staffing: (1) a lack of training and experience in treating PEH with buprenorphine and (2) a shortage of clinicians who provide low-barrier buprenorphine treatment. One clinician reported: “*There are not enough providers who do the low-barrier start. There’s not enough there should be more*.” (Clinician).

Many participants reported that stigma toward PEH with OUD persists among clinicians, and these negative attitudes may discourage them from offering buprenorphine. They reported observing these attitudes firsthand and hearing about them from patients. Related to the theme of *health care-related stigma*, one clinician commented: “*I do think that stigma still plays a large role in access to buprenorphine even within pharmacies, unfortunately, in terms of a pharmacist’s [un]willingness to engage with clients experiencing homelessness*….” (Clinician).

Participants expressed concerns about challenges in initiating XR-buprenorphine, noting that the medication, which was available at the time of the interview, is administered in fixed doses (i.e., 100 mg or 300 mg) that cannot be adjusted. Clinicians viewed the standard protocol as inflexible and struggled to adhere to it (Q2).

### Institutional-Level Barriers

Clinicians reported that the larger social context of pressure from medical boards and federal agencies (e.g., the Drug Enforcement Agency [DEA]) could stigmatize and discourage prescribing of buprenorphine. They felt that regulatory restrictions on buprenorphine prescribing and dispensing caused fear among providers, discouraging them from treating patients with the medication. Related to the theme of *regulatory factors*, one clinician reported: “*And so we get a lot of pressure as pharmacists to ensure that we are not engaging in prescriptions that are not necessarily legal prescriptions [or] don’t have a clear medical indication. [A]nd we get those from both the Board of Pharmacy and from the DEA. So, I think this fear has been really instilled with us…And so we get these messages that these are not good medications…We should not [use] them. But I think it creates a lot of fear...we should be really actually encouraging buprenorphine, if we’re having an opioid epidemic.*” (Clinician).

State regulations limiting clinical pharmacists’ authority to prescribe buprenorphine were another barrier to expanding access. A participant described that collaborative practice agreements allow clinical pharmacists to prescribe and refill buprenorphine prescriptions empowering them to practice as an extension of other healthcare care professionals. Related to the theme of *access*, the participant noted that provider capacity and treatment access barriers could be addressed by pharmacists.

“…*[With] a collaborative practice agreement … pharmacists can get out there and start managing buprenorphine and OUD. Whether it is inductions or whether it is just managing all the refills for the people that are currently on it, just making sure they’re getting their refills appropriately on time. Tele-BUP, like I said, has increased some of the provider availability, but there’s still barriers, there’s still access issues. Even with them and there can sometimes be long waits. Provider availability, I think, is still one big thing, and I see so many people coming here that don’t have prescriptions, and I keep thinking, if we just had a collaborative practice agreement*.” (Clinician).

Participants noted that lack of insurance and identification requirements hinder access to OUD treatment for PEH. Pharmacies often require valid identification to pick up prescriptions, which many PEH cannot provide. Also related to the theme of *access*, one clinician reported: “*Insurance status is a barrier and documentation, or identification is a barrier*.” (Clinician).

Many participants reported that care continuity challenges hindered access to and maintenance of buprenorphine treatment for PEH. They described that both patients and clinicians had too many steps to complete to deliver, access, and maintain treatment (Q3). Related to the theme of *access*, these multiple steps often disrupt care, particularly because patients must see many different clinicians with insufficient communication among them.

“*That’s the challenge when working with somebody who’s interested in getting off heroin because it’s always been frustrating –when somebody is ready for a change, and you can’t immediately get those referrals and resources together, like you’ve lost, because the following day they may not be ready for a change*.” (Support Service Administrator).

### Provider Recommendations for the Expansion of Buprenorphine Treatment for People Experiencing Homelessness

Participants recommended several strategies to expand buprenorphine access (see Table 2), including increasing knowledge about buprenorphine among both providers and patients. They emphasized the need for ongoing education for healthcare providers (i.e., nurses, primary care physicians, emergency medicine physicians, pharmacists) on advances in OUD treatment, including new drug formulations, to address the fentanyl epidemic. Addressing barriers related to themes of *knowledge and information*, participants suggested expanding educational programs on micro-dosing protocols [15] and XR-buprenorphine [(Q1)] (Table 2). They also emphasized training healthcare providers in harm reduction principles to reduce stigma and barriers to buprenorphine access (Q2). Common forms of stigma faced by PEH with OUD in healthcare settings include “enacted stigma,” where providers offer suboptimal care due to discriminatory attitudes, and “anticipated stigma,” where PEH avoid or delay healthcare due to past experiences of stigmatization [16].

**Table 2:** Provider quotes supporting recommendations for expanding buprenorphine accessibility

Quotes	Barriers targeted
(Q1) “…[dept. of behavioral health services] has been doing some great work around creating bubble packs for micro dosing for people who have been struggling with the withdrawal process. And particularly fentanyl users where they have a seven-day titration protocol or a three- day titration protocol where the idea behind it is that you slowly ramp up the amount of buprenorphine in your system as you slowly ramp down the amount of fentanyl. And then you avoid the withdrawal process, but you have to be very health literate in a lot of ways to accomplish it” (Clinician)	Knowledge and information
(Q2) “… training in what harm reduction for healthcare professionals, so that they are able to be more compassionate and empathetic when patients who use drugs come to them.” (Support Service Staff)	Staffing Health care-related stigma
(Q3) “I always have clients who say, ‘well why can’t I do it now? I’m here now. Why do I have to come back?’ …I think [having to adhere to an induction protocol] is a big barrier. Education on what the injectable looks like, and information on the different stages, and the length of time for when things will kick in, and how quickly it will kick in. Just overall injection medicine education is needed.” (Clinician)	Knowledge and information Concerns about medication and its administration Access
(Q4) “The way that we have it set up now, and it’s just not working as well as it could, is that at each SIP [Shelter in Place Hotel] there’s a room that’s designated for medical staff. And those rooms could operate more effectively as mobile clinics. Maybe there could be a van sort of situation so that the infrastructure of the clinic can be exported in a more mobile way. Cause you don’t necessarily need like a concrete building to do a UTOX [urine toxicology screening] or to give a Sublocade injection. You just need certain things, like a safe place to sit down, some supplies, a refrigerator, that sort of thing.” (Clinician)	Regulatory factors Access
(Q5) “[COVID] provided this unique opportunity where the providers were going in [to SIP hotels] and they had this group of patients that were using opioids, and probably some of the higher risk patients were getting funneled into the SIP sites” (Clinician)	Regulatory factors Access

Participants suggested that increasing patient education in clinics, along with peer-to-peer education and outreach in non-clinical settings, could help address barriers of misinformation and negative past experiences. Peers conducting street outreach, in particular, could be especially effective in building trust and providing information and encouragement to individuals with OUD in initiating buprenorphine treatment [17]. Additionally, participants noted that PEH with OUD can also benefit from a greater understanding of buprenorphine treatment formulations, including induction procedures and maintenance requirements (Q3).

Participants highlighted expanding low-barrier buprenorphine models and improving service delivery efficiency as key institutional-level strategies to enhance access. They noted that the San Francisco Department of Public Health has successfully operated a street medicine-based buprenorphine program for PEH since 2003. To improve care continuity, they suggested expanding this program and integrating buprenorphine treatment across various community settings, including opioid treatment programs, pharmacies, street-based outreach, harm reduction services (e.g., syringe access), and mobile van services (Q4).

Participants noted that the daily challenges of homelessness are a major structural barrier to buprenorphine use. They recommended increasing access to permanent housing as a public policy strategy to improve buprenorphine treatment engagement, stabilization, and adherence. This was supported by the observation that buprenorphine treatment increased during the COVID-19 pandemic when PEH were rapidly and temporarily housed in shelter-in-place hotels where onsite buprenorphine reduced barriers to filling prescriptions (Q5).

## Discussion

Providers identified barriers and recommendations for buprenorphine treatment for PEH with OUD at the patient, clinic, and institutional levels. Patient-level barriers included knowledge gaps, concerns about the medication, and challenges due to homelessness. Clinic-level barriers focused on staffing and health care-related stigma. Institutional barriers involved state regulations and access issues. Laws limiting clinical pharmacists’ ability to prescribe buprenorphine restrict low-barrier treatment access in community settings [18], while ID requirements for medication and care continuity challenges further hinder treatment. Additionally, homelessness-related resource shortages raised concerns about managing withdrawal before starting buprenorphine and made long-term medication storage difficult due to theft risks [15, 19].

Patient-level barriers to buprenorphine align with prior studies of individuals with OUD in and out of treatment. Negative experiences with buprenorphine, such as precipitated withdrawal from concurrent opioid or non-prescribed buprenorphine use, have been cited as barriers to starting treatment [20, 21]. A lack of knowledge about buprenorphine formulations is also a barrier to initiating treatment [20]. Further, two other studies of patient attitudes toward XR-buprenorphine found patients may be skeptical about its effectiveness and have concerns about its negative side effects, duration of the effects [22], and irreversibility [23]. However, a study of PEH in an outpatient opioid treatment program tailored to their needs found that some preferred XR-buprenorphine because it eliminated the inter-dose fluctuations of blood levels associated with sublingual buprenorphine [24]. Results from our study highlight the need for interventions to address limited knowledge about buprenorphine formulations among PEH and negative attitudes toward treatment [23, 25]. Stigma reduction interventions targeting healthcare professionals, such as exposure to visual campaigns and narrative vignettes, have been shown to effectively reduce negative attitudes toward individuals with OUD [26].

A national study on prescription trends found that the 2022 elimination of the X-waiver, a federal requirement for healthcare providers to complete training and obtain DEA authorization to prescribe buprenorphine for OUD [27], did not significantly increase buprenorphine prescriptions [28]. This suggests that removing the X-waiver alone is insufficient to improve buprenorphine access or address the overdose risk among PEH. Additional measures are needed, including low-barrier, community-based access to reduce stigma and address this urgent need [29]. Research shows that partnerships with community-based organizations offering mobile, van-based, shelter-based [30], and harm-reduction program-based buprenorphine can alleviate patients’ distrust of healthcare systems and improve buprenorphine acceptance [31, 32]. One study of a low-barrier, street-based medicine buprenorphine program for PEH argued that metrics for success should include intermittent buprenorphine use and reduced opioid use, rather than the standard focus on consistent buprenorphine use and abstinence from nonprescribed opioids [7]. Moreover, COVID-era regulatory changes, such as eliminating prior authorization for XR-buprenorphine and allowing telehealth-based buprenorphine initiation, expanded access [33–36]. These adaptations in service delivery could signal improved alignment between clinician and patient goals for buprenorphine treatment.

Research on buprenorphine initiation in clinical settings has highlighted treatment retention as a key challenge, especially for PEH [29]. Our findings build on this by identifying continuity issues across community settings, including street-based, low-barrier buprenorphine access programs, community pharmacies, and brick-and-mortar primary care clinics. Recent studies have recognized pharmacists as key to ensuring treatment continuity, with community pharmacies seen as critical sites for expanding buprenorphine access [37]. Homelessness, especially with fentanyl as the primary opioid, complicates care continuity. PEH with OUD who start and stop buprenorphine treatment risk recurrent episodes of precipitated withdrawal in street settings without access to basic sanitation and safety [38].

### Limitations

## Conclusion

Housing vulnerability increases risk for opioid overdose, underscoring the need for accessible medications for PEH with OUD [1]. We identified key barriers to buprenorphine access at the patient, clinic, and institutional levels. Recent healthcare policy changes, such as the repeal of the DEA X-waiver for prescribing buprenorphine, expanded telehealth access for treatment initiation, the Mainstreaming Addiction Treatment Act (allowing pharmacists to prescribe buprenorphine), and federal funding for OUD treatment, may improve access for marginalized populations, like PEH. These policy changes could expand low-barrier routes for initiating and maintaining buprenorphine, including XR-buprenorphine, which shows promise for improving care continuity [9]. Future research should examine the impact of these policy changes on care access and clinical outcomes for PEH.

## Data Availability

Data is available on request to preserve participant privacy.
